# Naturalness and the Legitimacy of Thoroughbred Racing: A Photo-Elicitation Study with Industry and Animal Advocacy Informants

**DOI:** 10.3390/ani10091513

**Published:** 2020-08-26

**Authors:** Iris M. Bergmann

**Affiliations:** School of Geosciences, The University of Sydney, 2006 New South Wales, Australia; iris.bergmann@sydney.edu.au

**Keywords:** thoroughbred welfare, equine welfare, naturalness, thoroughbred racing, photo-elicitation, animal welfare, animal protection, horse-human relationships, human-animal relations

## Abstract

**Simple Summary:**

The international thoroughbred industry is concerned about the public’s perception of racing. Therefore, the industry’s priorities are to address the publicly most visible and known welfare violations. However, common day-to-day racing practices also impact thoroughbred welfare. In this study, key industry informants and animal advocacy informants were interviewed to find out how they view common racing practices. For the interviews, photographs of thoroughbreds on race day were used, which the informants were asked to describe. Results show industry informants often naturalise, normalise, downplay or ignore the horses’ expressions, the impact of handling on the horse and the use of equipment. The animal advocacy informants tend to describe a horse whose nature is violated. In conclusion, the industry informants show limited interest in addressing common racing practices, and this places thoroughbred welfare at risk. Both groups of informants have different ideas about what is natural and what that means for thoroughbred welfare. With society’s understanding of welfare and of racing practices growing, the racing industry may be increasingly questioned about common racing practices. This article discusses the notion of naturalness in more detail and how it can be used to advance thoroughbred protection.

**Abstract:**

The idea of what is natural has particular relevance in the thoroughbred racing and breeding discourse. It guides breeding regulations; influences how the thoroughbreds’ behaviour is perceived and has implications for husbandry, handling, training and racing practices. This study investigates how key industry and animal advocacy informants based in the US, Australia and the UK conceptualise naturalness within the context of common racing practices that potentially impact the horses’ welfare. The informants were interviewed using semi-structured interviewing and photo-elicitation. Four common images of thoroughbreds on race day were presented to elicit the informants’ responses. Differences emerged between how the two groups tended to describe the images and the role naturalness played in their conceptualisations. The findings were analysed using an updated version of the Layers of Engagement with Animal Protection developed by Bergmann to situate the informants’ conceptualisations of naturalness within the wider thoroughbred protection discourse. In conclusion, the industry informants tended to defend the status quo of common racing practices. They tended to naturalise and normalise these practices and downplay their welfare impact. This poses risks for thoroughbred welfare, which are amplified by misrepresentations of what is natural. With the public’s understanding of welfare and racing practices growing, racing’s legitimacy may be further questioned. Opportunities to leverage the potential of the notion of naturalness for thoroughbred protection are discussed.

## 1. Introduction

Concern about the public’s perception of thoroughbred welfare is reverberating throughout the international thoroughbred racing industry. In 2019, thoroughbred welfare was nominated as the theme of the annual conference of the International Federation of Horseracing Authorities (IFHA), a body created to harmonise the rules of its 59 member countries for breeding, racing and wagering. Agenda items included the question of how the racing authorities of its member countries define welfare and how they should respond to the changing “consumer and political environment” [1]. Bergmann [2] studied the conceptions of thoroughbred welfare held by key individuals in governance and senior administrative and executive roles in the international thoroughbred industry. Three main groups of welfare issues emerged: injuries and deaths on the track, use and overuse of drugs and medication and the retirement of thoroughbreds. The informants’ attention is focused on the most egregious and abusive practices, those that are most visible and have been centred in the public discourse. Yet, these welfare issues are only the proverbial “tip of the iceberg”. Animal advocacy informants in the same study additionally identified routine training and husbandry practices, human-horse interactions and the “everyday life of horses” as “where the real welfare issues are” in thoroughbred racing [3]. These are issues discussed in the general equine welfare literature and include topics such as housing [4,5,6,7], feeding [8,9], equine behaviour [10], equine emotions [11], equine welfare assessment [12,13], the application of equipment [14,15,16,17,18,19,20,21], equine learning and training [22,23], the impact of equine activities on the horse [24], human handling during various forms of human-horse interactions [25,26], impacts of riding on behaviour and welfare [27,28,29,30], horse-human relationships [31,32,33,34] and people’s ability and inability to recognise behavioural signs of equine distress and pain [35,36,37,38]. A theme that unites these issues and that allows one to make assessments as to the welfare impact is naturalness, i.e., what is natural for the horse and what is in the horse’s nature in relation to their species-specific, as well as individual, physiological; emotional; cognitive; social and behavioural characteristics, abilities and boundaries. These welfare issues do not appear to be recognised by the thoroughbred industry as critical for the integrity of racing, nor for how the industry is perceived by the public [2,3,39].

The general racing participants’ discourse about what is natural is based in the horse’s emotional realm and encapsulated in the phrase the horse “loves to race” [2]. This view is upheld even in the presence of horse behaviour that phenomenologically does not seem to support this idea [2] (p. 130). There is also a biologically based claim that horses choose to run or race if given the opportunity to move freely. However, if given the choice, horses spend the majority of their time foraging and grazing [40,41]. The time horses in the wild spend moving mostly involves walking, with some trotting and cantering, but rarely galloping [42]. Equating this with a highly regimented training regime where horses are asked repeatedly to perform at and beyond their natural limits appears flawed (see more in Section 4.4.1). 

In the sphere of thoroughbred breeding, the most significant attribution of natural is situated in the biological realm. The thoroughbred industry vehemently protects conception by “natural” means to produce an “eligible foal” [43] (pp. 46–47), which is unique to this industry [44] (p. 173). Breeding practices, however, are far from natural and highly invasive for both mare and stallion [44] (p. 183), and the insistence on natural breeding is less about protecting thoroughbreds but often seen as a means to protect investments.

What is considered natural influences how the thoroughbred is handled and trained; it influences husbandry practices and breeding regulations. Yet, the idea of what is natural is riddled with contradictions and inconsistencies considering the controlled and confined conditions racehorses live in, the amount and types of medications and drugs and surgical procedures used to breed, sell, train and race thoroughbreds, the human-determined pathway of their existence [44]. As McManus et al. [44] (p. 175) state, there are conceptual challenges for the industry. In this article, it is argued that what is at stake is the legitimacy of thoroughbred racing based on the treatment of the horse and that this treatment is influenced by perceptions of what is natural for and about the horse.

In light of the above, the aim of this study is to explore how key informants of the thoroughbred industry conceptualise naturalness and what is natural for the thoroughbred in racing, how this impacts their perceptions of common racing practices on race day, which potentially impact the horses’ welfare, what implications this has for thoroughbred welfare and how the industry is positioned to respond to society’s evolving attitudes to animal welfare. The aim of this study is also to explore the views of animal advocacy informants to canvas the diversity of perspectives that influence the development of future thoroughbred protection regimes. The goal for the study is to elucidate the role of conceptualisations of naturalness and to explore the potential of the applications of this concept for the protection of thoroughbreds and, by implication, other animals. Naturalness in this study is treated as a lens through which all aspects of the thoroughbred’s life are viewed. 

## 2. Competing Conceptions of Naturalness

Recently, a growth in interest in the concept of naturalness and its application can be observed [45,46,47,48,49]. Naturalness is generally seen as one of the three dimensions to describe animal welfare, the other two being basic health and functioning and affective states [50]. Fraser [50] summarises that those engaging in the welfare discourse and expressing a concern for naturalness refer to the ability of animals to live reasonably natural lives by carrying out natural behaviours, by having natural elements in their environment and a respect for the nature of the animals themselves. Animal welfare scientists, however, generally apply naturalness to animal behaviours only [48,49]. Yeates [48] appears to be the first to develop a definition for naturalness and a way of assessing it, from this narrow point of view. He suggests defining natural behaviour as being “unaffected by man (sic)”, and the naturalness of an animal’s behaviour can be assessed in terms of its similarity to an equivalent unaffected wild animal. This definition of natural behaviour has been criticised as too narrow by Gygax and Hillmann [45] and as being irrelevant for our understanding and measuring of welfare by Browning [51]. Others outside animal welfare science like Hadley [46] argue for a holistic and representational definition of naturalness that considers how citizens view naturalness. 

Clark et al. [52], in reviewing 80 studies published between 1995 and 2015, found that naturalness is central to public attitudes and concerns in relation to animal welfare. They [52] (p. 462) summarise that people find naturalness is important for the physical and psychological wellbeing of animals, and the hampering of natural behaviour is seen as having a negative impact on the animals’ overall health. The tendency for people to value naturalness is confirmed by subsequent studies [53,54,55]. People compare a variety of aspects to what is natural, including animals having enough space and associated freedom to behave according to their natural instincts, having access to the outdoors and to unadulterated feed [52] (p. 46), and they refer to freedom of movement and a natural lifespan [53]. People consider eating pelleted feed as being against the animal’s nature [56] (p. 195). They are repelled by and concerned about practices they consider to be unnatural, such as the breeding of farm animals using artificial insemination [55] (p. 44) [57] (p. 30), and they oppose zero-grazing and cow-calf separation due to the loss of naturalness [54]. Furthermore, Robbins et al. [58] found people generally prioritise naturalness over emotional states. They explain, “a chimpanzee living a natural life with negative emotions was rated as having better welfare than a chimpanzee living an unnatural life with positive emotions”, and for “chimpanzees with positive emotions, those living a more natural life were rated as happier than those living an unnatural life” [58]. It appears that naturalness is a lens used by people when making assessments about what a good animal life is. The range of aspects that people relate to naturalness indicate that they conceptualise naturalness in holistic terms. 

In the equine welfare literature studying horse people’s attitudes to equine welfare, naturalness also features. Thompson and Clarkson [59] found that it is important for horse owners to determine whether their horses’ (natural) social and behavioural needs are met. Horseman et al. [60] studied the perception of welfare of a range of stakeholders in the equestrian industry in the UK, including owners, riders and coaches. They found participants addressed naturalness by referring to natural behaviour and the horse’s natural needs. They also found “the emotional experience of the horse emerged as an important component of welfare… and the interviewees made a link between the emotional well being of the horse and the provision of ‘natural’ needs” [60] (pp. 9–10). They suggest that, despite intuitively seeing aspects of naturalness as important, the interviewees found it hard to articulate. These findings are reflected in studies of the thoroughbred industry. Butler et al. [61] found that people professionally involved with the care of racehorses in the UK believe “keeping the horses’ lives as natural as possible” to be part of a “best-life” scenario. However, some also saw situations where the risk of injury outweighs the benefits, as for example, when providing a shared turnout for horses that they believe bears the risk of injury due to horses kicking each other. The authors state “[w]hat constitutes ‘natural’ for a racehorse may be difficult to define”, but they indicate that it includes freedom of movement and choice [61]. In the horse world, the idea of what is natural is also referred to in the horse-training technique “natural horsemanship” [62]. However, interestingly, in the relevant studies cited above, references to natural horsemanship are not made. In terms of racing specifically, although some individual owners and trainers may advocate aspects of natural horsemanship, it does not play a role in the thoroughbred industry discourse [2,3,39,61,63]. 

Based on the studies discussed above, it appears that, overall, interest in the concept of naturalness is increasing, and this is likely to have implications for the discourse of thoroughbred welfare in the thoroughbred industry.

## 3. Materials and Methods

### 3.1. Scope of This Study

This research is part of a larger exploratory study that investigates the intersection of thoroughbred protection and sustainability in the international thoroughbred industry [3]. As part of that larger study, Bergmann [3] developed a theory of interspecies sustainability. This current article focuses on one aspect of this theory, namely naturalness [3]. While thoroughbred breeding and racing are deeply entwined, the focus in this article is on racing. There are differences in regulations and risk factors between racing jurisdictions, but these are not considered in greater detail unless they contribute to the understanding of a particular argument. It is also recognised that the industry is working towards national and international harmonisation of the Rules of Racing [64]. Therefore, the thoroughbred racing industry can be referred to in general terms, whilst also considering relevant national differences emerging in this study [3]. Both industry and animal advocacy informants were invited to participate as part of a symmetrical research design to include the diversity of views likely to influence the direction of thoroughbred protection measures and for triangulation (for more on triangulation and other procedures for trustworthiness, see Appendix A). The hypothesis was that there are differences in how the two groups of informants conceptualise naturalness and what is natural for thoroughbreds in racing and that this impacts their perceptions of common racing practices on race day. The study aimed to consider events that were not necessarily representative of all events on race day but those potentially attracting attention because of possible impacts on the horse’s welfare.

### 3.2. Informant Recruitment and Response

Thirty-seven administrative and regulatory bodies of the thoroughbred industry affiliated with the IFHA and based in Australia, the UK, Ireland, New Zealand, the US and Hong Kong were contacted via email. Sixteen did not respond after follow-up emails, and thirteen declined. Eight industry participants from seven organisations, and one individual at the time of the interview not affiliated with any organisation, from Australia (3), the US (5) and an international body (1), agreed to participate. Animal advocacy organisations whose websites published information about thoroughbred racing, indicating some expertise on thoroughbred welfare, were contacted. No such organisation could be identified for Ireland or Hong Kong, but thirteen in Australia, New Zealand, the UK, US and one international organisation were contacted. One organisation declined, stating they lacked the expertise, three did not respond, while seven based in Australia (3), the UK (2) and the US (2) agreed to participate, bringing the total number of informants to sixteen.

The industry informants were in senior and executive roles in their organisations, in regulation, general management, development, marketing and communications, and as a board member. The organisations included breeders, racetracks, jockey clubs, regulatory bodies and national and international bodies. The informants’ backgrounds included training and experience as veterinarians; in science, agricultural and applied economics; law; management; insurance and broadcasting. All had a long history of involvement with racing. Some were, or had been, owners or breeders of racehorses. The animal advocacy informants were employees of their organisations—some in executive roles, others in scientific or animal welfare roles—and, again, others were affiliated consultants. It can be assumed that the informants were “central actors whose individual [perspectives] matter" [65] (p. 194).

The difficulty in recruiting racing industry participants for research that is associated with thoroughbred welfare has also been experienced by Butler et al. [61,63]. Given the controversy and tensions surrounding welfare in racing, the number and organisational roles of industry informants who agreed to participate can be considered successful (see also Bergmann [3]). 

The University of Sydney Human Research Ethics Committee (HREC) approved the protocol for this study, Project No.: 2016/019, on 22 January 2016.

### 3.3. Data Collection and Analysis

Semi-structured interviews were conducted via telephone and Skype between February and August 2016. The interviews included semi-structured interviewing and photo-elicitation. The units of analysis [66] relevant for this article included responses to three conventional verbal-only questions of the larger interview schedule and responses of the photo-elicitation phase. The three verbal-only questions were posed at the beginning of the interview, asking the informants what the thoroughbred represents for them, what they believed is the most natural (equestrian) activity for the horse and how they defined naturalness. Then, questions about thoroughbred welfare, sustainability in racing and the interface between the two followed, with the responses to these questions analysed previously [2,3]. Next was the photo-elicitation phase. The process used for photo-elicitation is described in Section 3.3.3.

The full interviews took approximately one hour, except in two instances, when they took approximately 105 minutes. One of these instances involved two informants of one organisation who requested to be interviewed together via telephone. In this case, the interviewer ensured that both informants had equal opportunity to respond. Both informants represented their perspectives with confidence and contributed independent ideas. Some converging of responses could be observed in a few instances, and that was considered in the analysis. Overall, their responses were situated within the range of the group of industry informants’ responses. Had both these informants represented more extreme perspectives simultaneously at any one time during the interview, this would have been considered and commented on in the analysis. This was, however, not the case. 

#### 3.3.1. The Photo-Elicitation Method

This study employed photo-elicitation using images of thoroughbreds on race day to elicit the informants’ responses. This served the following purpose: This study centred around the welfare and protection of thoroughbreds. For this, their lived experiences had to be foregrounded. Using photographs are one way of foregrounding their experiences and letting them “speak for themselves” [67]. Via their photographs, the thoroughbreds elicited responses in the human actors, the informants of this study. These responses were expressions of how the informants saw the experiences and the welfare impacts of common racing practices on the thoroughbreds. Photo-elicitation gave the informants the opportunity to draw on a rich repertoire of their cognitive processing to interpret what it was that they saw [68]. The above is further discussed below.

Photo-elicitation interviewing is one of many visual research methods used in the social sciences [69]. In this interviewing technique, researchers use photographs during the interview and ask the participants to comment on them. The photographs can be drawn from image banks and can be researcher- or participant-generated [70]. Photo-elicitation was initially applied in anthropological research, with Collier [71] often cited as the first published study [72,73]. It has subsequently been used in anthropological and ethnographic research [74,75]; in sociological [76], educational [77,78,79] and psychological research [80] and in organisational [81] and health-related studies [82]. More recently, it has been used in research contexts broadly related to the thoroughbred industry. For example, Ward and May [83] explored the mental images veterinary students held of the veterinary profession; Mills et al. [84] explored farmers’ and veterinarians’ perceptions of dairy cow welfare and others researched the interface of land conservation, agricultural practices and local knowledge [85,86,87,88]. Two of these broadly related studies [82,85] used photo-elicitation to compare the perceptions of two groups of participants, similar to this current study. While Ward and May [81] supplied photographs drawn from image banks to present during the interview, the other four studies involved their participants in taking photographs that were then used for interviewing.

This study is situated in the field of animal studies, a subdiscipline of the social sciences that began to emerge during the mid-1990s [89] (p. 308). As O’Sullivan et al. [90] (p. 362) point out, animal studies is “underpinned by a pro-animal theoretical frame, meaning the research is focused on progressing the wellbeing of animals, much as the study of human rights is typically focused on advancing rights, rather than say, enhancing opportunities for genocide”. Animal studies draws on the actor network theory (ANT), establishing that nonhuman animals are actors and to be considered as such in the research process [91]. Animal studies scholars are developing methods that take account of the nonhuman as an actor and participant in the research process. They attend to the “lived experiences of animals and the nonhuman side of human-animal relations” [92] (p. 769). Visual methods are used as one way of centring the experience of the animals and of giving the animals a voice in the research [67,93]. 

For the current study, the photographs were taken by the researcher capturing “common” scenes on race day, centring the experience of the thoroughbred (see Section 3.3.2). Photographs have the potential to trigger memory and give access to new understandings of memories [94] (pp. 5,6). Thus, it was expected that informants would draw on their own experiences with thoroughbreds and the racing context, potentially eliciting new meanings in relation to the thoroughbreds’ experience and their welfare and establishing new connections between the elicited phenomena. Using photographs was expected to ground the informants’ thinking in the thoroughbreds’ experiences as captured in their behavioural and mental expressions and in relation to what else can be seen in these photographs. It has been established that photographs serve as stimuli yielding qualitatively different kinds of information than do interviews that rely on the verbal mode only [68,72]. This methodological approach therefore augments the verbal-only interview phases. 

Using photographs of thoroughbreds who were the subjects of concern, visualising their lived experiences of common racing practices also carried an emancipatory element. It sought to empower the most disempowered and vulnerable in the study context. This has been the underlying objective of many of the photo-elicitation studies in the social sciences [73]. The researcher taking and selecting the photographs to match the requirements of this research context and the research aims (Section 1) was considered the next-best way to let the thoroughbreds “speak” for themselves and of their lived experiences in racing [67,93]. 

#### 3.3.2. Image Creation and Selection

The study aimed to use images that were not necessarily representative of all events in thoroughbred racing on race day but those potentially attracting attention because of possible impacts on the horse’s welfare. The images used for photo-elicitation had to be relevant for the research context and the aims of this study (Section 1). They had to depict some kind of observable emotional or behavioural response of the thoroughbred that provided interpretive space for the informants. The images had to be within the realm of what the literature cited in Section 1 has identified as compromising horse welfare and, also, within the realm of what is considered common on race day. Images that can fairly be described as “benign” and leave little room for interpretation of any potential welfare impact of common racing practices—for example, horses grazing—were not relevant for this research. Images more directly alluding to severe or potentially severe harm—for example, horses falling—were also not relevant for this research. 

The process for creating and selecting the images began with taking 998 digital photographs at race meetings at three different locations. Of those, 364 photographs depicting thoroughbreds at various stages before, during and after the race were selected. Photographs depicting dominantly people or scenery, or horses too distant, were eliminated. The selection was then narrowed to eight images and, finally, to four images, as per the following six criteria:

- The thoroughbred was to be the central focus, filling all or most of the image frame, with some contextual background where relevant.
- The scene, environment, equipment used and handling by any humans should generally be considered “common”.
- The photographs were not to depict any extreme responses of either human or horse.
- They should however depict some behavioural response that offered interest and room for interpretation.
- The photographs had to be of good quality in terms of framing, focus and exposure.
- Each image had to depict a different aspect of interest and context.

**Figure 1:**
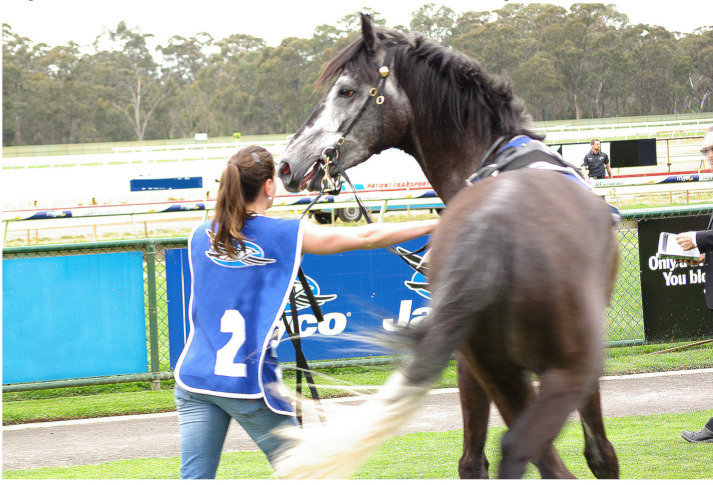
Image 1 for photo-elicitation interview.

The full interview involved six photographs for photo-elicitation; however, only four of these images were used for the analysis in this article. These four images depict individual thoroughbreds on race day. The other two images depict thoroughbreds in alternative settings and contexts that were beyond the scope of this article. In terms of digital image processing, sharpening, adjusting exposure, contrast and cropping to centre the areas of interest without change to the overall appearance or actual event was deemed acceptable. For publication in this article, advertising has almost completely been removed, and recognisable human faces have been pixelated. The following four photographs were included in this study: 

Image 1 (Figure 1) shows a full-body view of a saddled thoroughbred led by a handler. The thoroughbred, as well as the handler, show a distinct behavioural response.

**Figure 2:**
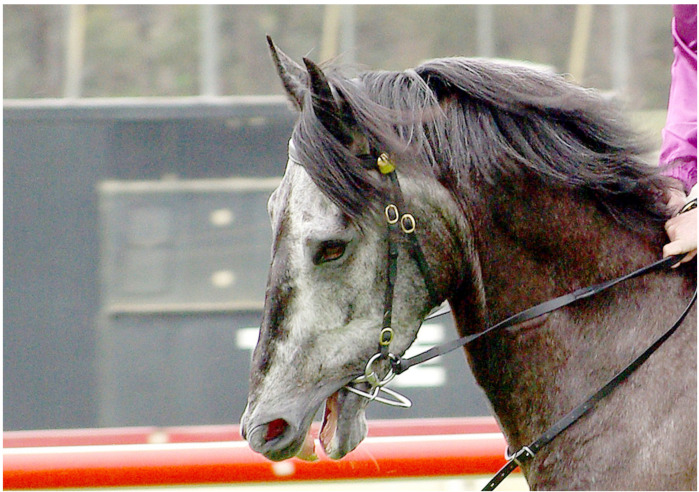
Image 2 for photo-elicitation interview.

Image 2 (Figure 2) shows a moving thoroughbred’s head close up, as well as part of the jockey’s hand and arm. The jockey holds close contact with the reins, and the horse’s mouth is open.

**Figure 3:**
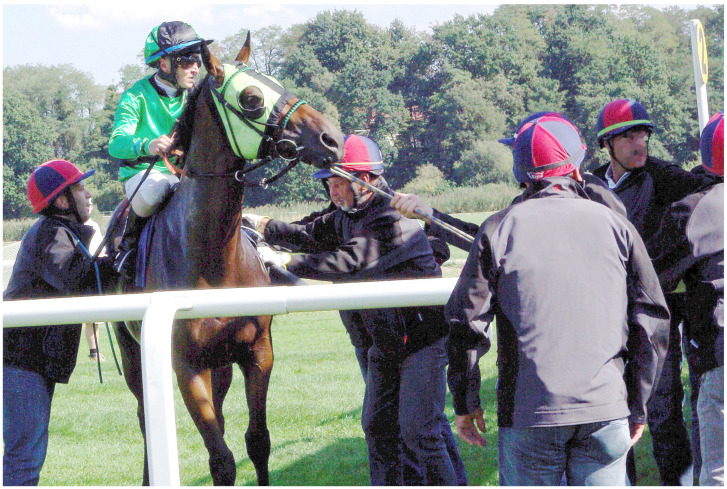
Image 3 for photo-elicitation interview.

Image 3 (Figure 3) shows a thoroughbred almost in full, with a jockey on his back, with six handlers close by, some touching the horse and some holding ropes attached to the horse. Handlers and horses show intent.

**Figure 4:**
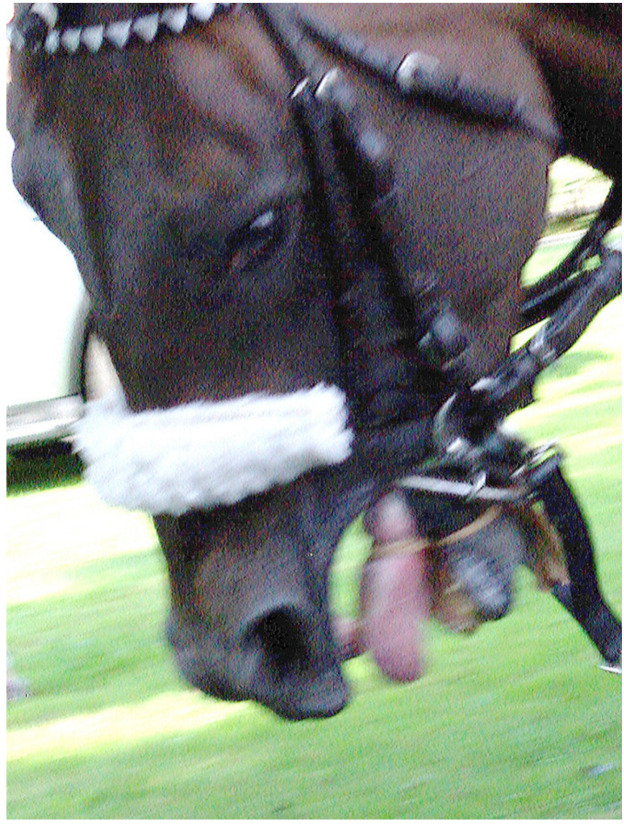
Image 4 for photo-elicitation interview.

Image 4 (Figure 4) shows a head of a thoroughbred close up, bridled and on a lead rope, head lowered, mouth opened and tongue and tongue-tie visible.

**Figure A1:**
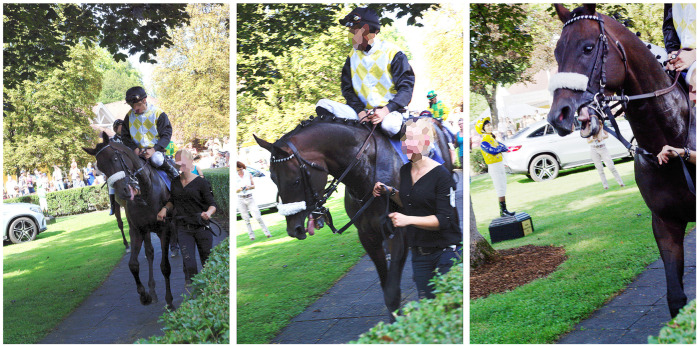
Images 1–3 of eight images of a thoroughbred with a tongue-tie taken on race day while the horse was led past the photographer/researcher in the mounting yard.

**Figure A2:**
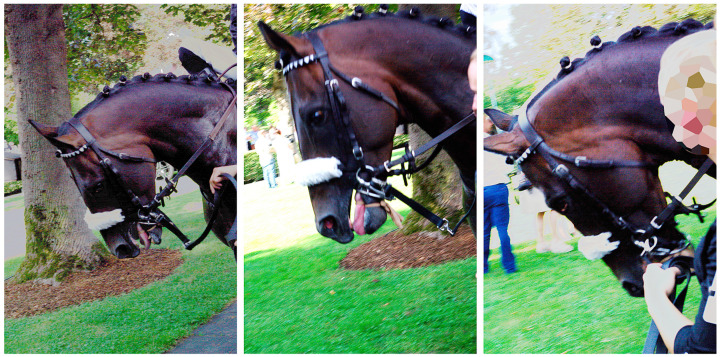
Images 4–6 of eight images of a thoroughbred with a tongue-tie taken on race day while the horse was led past the photographer/researcher in the mounting yard.

**Figure A3:**
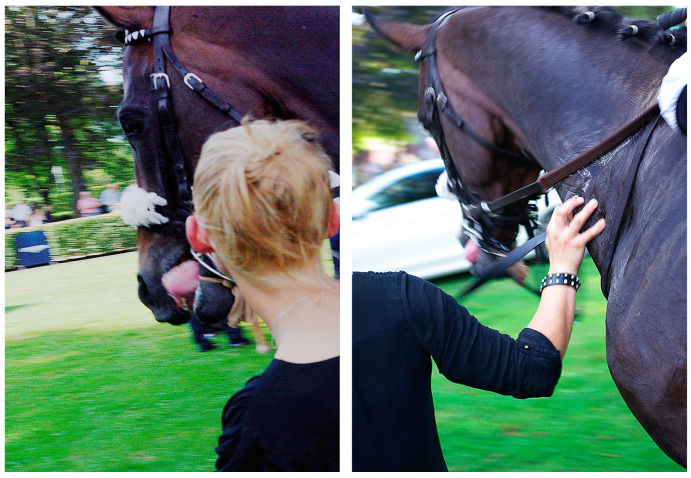
Images 7 and 8 of eight images of a thoroughbred with a tongue-tie taken on race day while the horse was led past the photographer/researcher in the mounting yard.

Image 4 required significant adjustment to the focus and exposure and was included, as this is a rare image capturing the tongue-tie and its impact on the horse while at work. Indeed, several informants made comments to the effect that the tongue-tie is rarely visible in this manner. The researcher took eight photographs. The present image was selected, because it shows the tongue-tie and the horse’s response but it does not show as severe a response as some of the other images, which might be considered uncommon, because still images of this kind are rarely publicly seen (see all eight raw images taken of the horse with the tongue-tie adjusted for light and contrast in sets of three, three and two images in Appendix B, Figure A1, Figure A2 and Figure A3)

#### 3.3.3. Photo-Elicitation Procedure

For this study, photo-elicitation interviewing involving the four images of race day scenes took between five to seven minutes, and approximately fourteen minutes for two informants, and it was embedded within an interview lasting between one and 1.5 h. For viewing, the photographs were uploaded to a website created temporarily for the purpose of this study. The hyperlink to that site was emailed to the informants prior to interviewing.

Before the photo-elicitation phase, the informants had already engaged with questions relating to thoroughbred welfare and aspects of sustainability (Section 3.3). When it came to the images, it was not the intention to conduct photo-elicited in-depth interviews, as is usually the case with photo-elicitation. The photographs were introduced to elicit spontaneous responses drawing on the informants’ personalised and emotive levels, experiences and memories (see Section 3.3.1). Therefore, the first of three questions stated: “Describe briefly what it is that you see, what comes to your mind first, your immediate reaction, please.” It could be expected that the contextual framework established by the preceding interview phase informed the photo-elicited responses. However, based on the requested spontaneity of response, it was expected that the informants would draw more on their personalised cognitive categories rather than on potentially stereotypical verbalisations of thoroughbred welfare. The question was devoid of nouns, adjectives or verbs that could lead responses. A second question followed to verify whether the images were considered to depict common scenes and events: “Is this a common thing that you see on the racetrack?” To provide opportunity to express any further thoughts, a third question was offered: “Anything else you would like to say in relation to this image?” In the case of questions from the informants or any prompts, again, no verbal reference points were given that could lead the informants’ interpretations.

The photo-elicitation and the semi-structured interview guide were pilot-tested with three participants unrelated to the informants of this study. Two participants of the pilot study had an equine veterinarian background and history of involvement with thoroughbred breeding and racing, and one participant was affiliated with an animal protection organisation. Based on the outcome of the pilot study, no changes to the instruments relevant for this study were required.

#### 3.3.4. Data Analysis

The interviews were audio-recorded, transcribed verbatim and imported into NVivo version 11 for coding and sorting. The transcripts were first coded deductively as per the questions; then, descriptive codes were applied. Themes were derived from the data inductively. The main analysis was based on inductive reasoning, since there was not enough existing knowledge about the phenomenon and what existed was fragmented [95]. Inductive reasoning moves from the specific to the general using observations, combining them into a larger whole or general statement [91].

The qualitative content analysis involves a “careful, detailed, systematic examination… in an effort to identify patterns, themes, assumptions, and meanings” [96] (p. 182). It was, in the first instance, a manifest analysis focussing on what the informants actually say, using the informants’ own words and describing “the visible and obvious” [66] (p. 10). It then moves into a latent analysis by extending into an interpretive level to uncover the underlying meaning and to identify themes [66] (p. 10) within the context of the research questions and aim. The themes are “an expression of the latent content of the [transcripts]” [97] (p. 107) to reveal the deeper layers of the responses. Two of the verbal-only questions asked directly about ideas of naturalness and what is natural. In the case of the third verbal-only question and the photo-elicitation, how the informants understand naturalness was inferred based on how they used ideas of the natural. This approach is based on cognitive theory and has been applied by other researchers [58]. 

**Figure 5:**
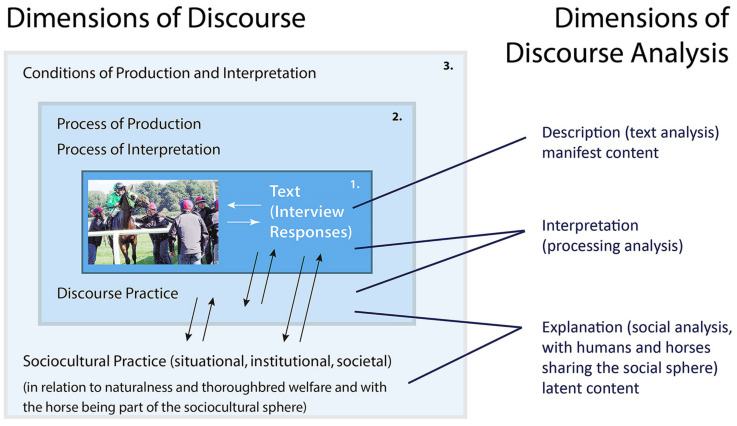
Dimensions of the discourse and discourse analysis (adapted from Janks [94] and Fairclough [96]) as they relate to the research process in this current study.

For the analysis of the photo-elicited responses, discourse analytical procedures as outlined by Janks [98] were adopted. Janks analysed images and related commentary applying Fairclough’s [99,100] three-part analytical model (Figure 5). This model accounts for the inherent nonlinearity of the analysis. It can be imagined as three boxes nesting within each other, each requiring a different kind of analysis: (1) text analysis (description), (2) processing analysis (interpretation) and (3) social analysis (explanation). 

The analysis does not necessarily follow one after the other but can move between all three. In the current study, the social analysis, which refers to “the bigger picture”, is represented by the discourse of naturalness at the meta-level within society at large (see Section 1 and Section 2) and in relation to what all this means for the thoroughbred. Thus, the naturalness discourse is the lens through which the social analysis is conducted. 

Finally, the analysis was deepened by the application of Bergmann’s framework of Layers of Engagement with Animal Protection [3]. For this study, this framework was updated to more explicitly include the notion of naturalness (see the updated version in Section 4.5.4). The informants’ conceptualisations of naturalness were then analysed, discussed and situated in relation to the Layers of Engagement (Section 4.5.4).

## 4. Results and Discussion

In the following, citations are assigned to the respective informants using acronyms—that is, TBI-n for thoroughbred industry informants and AAI-n for animal advocacy informants—with numbering of the individuals within each group from 1-9 and 1-7, respectively, to replace the value “n”. The informants’ responses describing what they see in the images relate to the temporal; spatial and intentional (when, where and what/why); descriptions and explanations of the horses’ mental and behavioural responses; human-to-horse interactions; descriptions and impacts of visible tack (bridle, bits, tongue-tie, reins and ropes); the environment for the horse overall and, in the case of one animal advocacy informant, horse conformation. The emphasis on each aspect varies by informant. Not all aspects are addressed for each image, and the two groups of informants place varying emphases on each aspect. 

The informants recognised the general location and moment in time depicted, with few variations. Importantly, what is depicted they considered to be common or “not uncommon” (TBI-9 on Image 1 and AAI-5 and TBI-2 on Image 4). Commenting on Image 3, AAI-5 (UK) stated: “Quite often, [handlers can be seen] around the horse, maybe not this many”, TBI-4 (Australia) said “it depends on the horse” and TBI-8 (US) conveyed a sense of resignation, having responded “you see this every single day”. There were variations, for example, by country in terms of the use of tongue-ties, as AAI-5 (based in the UK) stated, commenting on Image 4, “I wouldn’t say it was common [...] but we do see it from time to time”. Barakzai et al. [101] described the use of tongue-ties in thoroughbred racing in the UK as “commonplace” and found the proportion of starts with a tongue-tie is 5%. In Australia, it is reported to be 21.3% [102]. No industry informant from the UK agreed to participate in this study, so nothing can be said about a potential impact of the perception of common versus not-so-common use of tongue-ties on the industry informants’ conceptualisations. There does not appear to be any impact on animal advocacy informants’ conceptualisations. In principle, it can be stated that the informants of this study confirmed the photographs depict what can commonly be seen on racetracks on race day. 

Below, the results are structured to first present an overview of the two groups’ perspectives, then the themes as they emerge from each group’s photo-elicited responses. There are some inter- and intragroup variations, and negative cases and examples are presented. They can be explained within the broader context of the thoroughbred industry and the welfare discourse and, in particular, with the individual informant’s background. The need to preserve the anonymity of the informants limits discussions of their backgrounds. Relevant for this study are the breadth of perspectives and the emerging trends in the responses.

### 4.1. Overview

#### 4.1.1. Thoroughbred Industry Informants

Thoroughbred industry informants used assumptions of the nature of the thoroughbred as explanations for their mental and behavioural expressions. This nature was used to justify controlling mechanisms and practices they referred to in the photographs. There was also a tendency for industry informants to normalise and naturalise and, at times, downplay the thoroughbreds’ behavioural and mental expressions. This implies a naturalisation of the behaviour of the horse that transfers to a naturalisation of the entire process seen in the photographs, meaning a normalisation of the processes and procedures imposed on thoroughbreds in racing. The behavioural and mental expressions of the thoroughbreds in the photographs were seen more as a visual problem rather than a welfare problem. The thoroughbred was often portrayed as a willing and knowing participant, eager, excited and ready to race. The above is consistent with the industry informants’ view that racing is the most natural activity for the thoroughbred. In contrast to the above, where industry informants draw on the idea of the natural, they mostly did not regard the thoroughbred as natural anymore but as a product of human breeding. This is consistent with their overall low interest in the concept of naturalness in racing.

#### 4.1.2. Animal Advocacy Informants

Animal advocacy informants also used assumptions about the nature of the horse as an explanation for the thoroughbreds’ mental and behavioural expressions on race day. However, they tended to view the thoroughbreds’ assumed mental and behavioural predispositions as an explanation for why racing practices are not in the interest of their welfare. They mostly saw the thoroughbreds’ expressions as indicating stress, agitation, being disturbed and experiencing anxiety. They suggested the depicted racing practices are unnatural and have a negative impact on the thoroughbred. Animal advocacy informants tended to notice a broader range of factors impacting the thoroughbreds’ welfare by violating their nature, including a range of aspects of the overall environment and individual horse conformation. They tended to pay more attention and assign more welfare relevance to the horse-human interaction. The above is consistent with their view that racing is not the most natural activity for the horse; rather, they point out grazing, being with other horses and running as natural. In terms of a human-shared activity, leisurely trail riding at most comes close to being natural. As did the industry informants, the advocacy informants noticed a visual problem, albeit a very different one. They emphasised the lack of visibility of the breadth of the welfare issues to the public. Overall, animal advocacy informants described a more holistic view of naturalness, a view that is more consistent within itself and that demonstrates more consistency with ethological perspectives—that is, perspectives based on scientific studies of animal behaviours—in particular, as they occur in natural environments.

### 4.2. Themes Emerging from Industry Informants’ Photo-Elicited Responses 

Four key themes emerge from the industry informants’ responses to the photo-elicitation study.

#### 4.2.1. Naturalising and Normalising the Horses’ Responses to Racing Practices

Industry informants tended to describe and explain the horses’ mental and behavioural responses as being natural. For example, TBI-4 explained, commenting on Image 1: “When you get a horse in a parade ring at the races, there is a lot going on. Horses are naturally, their natural instinct is a flight or fight [...] the adrenalin is flowing there, he is sort of bouncing around and thinks what’s happening over there”. Similarly, TBI-5 commented on Image 3: “Perhaps the horse could have done with a bit more gate schooling, but you know what, it’s a thoroughbred. They sometimes just have their own way about things.” This normalising and naturalising culminated in the expression of industry informant TBI-7, having commented on Images 1 and 3: “I see a horse being a horse”. In justifying the horses’ responses as being natural and normal, any welfare concern was explained away. 

A notable exception is a response of industry informant TBI-9, commenting on Image 3, expressing concern and rejecting acceptability of what this informant saw:

“[This image] with the guys—one, two, three, four, five guys, six guys… Yeah, that, unfortunately, [...] I think that horse doesn’t want to go and there is probably a good reason why. [...] I wouldn’t be happy to see that [...] with them pulling him in. I hate to see when it’s, you know, there on the side they are using a tow rope in his mouth, pulling him to the gate. There is something wrong with that horse, he doesn’t want to go.” (Thoroughbred industry informant TBI-9)

This response represents the strongest stance in defence of the horse of any industry informant’s comment. TBI-9 did not elaborate, but considering the outlier position of this statement, it is more likely than not that this comment was triggered by the informant’s own experiences and memories (see Section 3.3.1).

#### 4.2.2. Downplaying the Impact and Role of Tack, Humans and Other Factors

In a number of instances, industry informants seemed to not only naturalise and normalise but downplay and trivialise the impact of racing practices. One strategy was to ignore what can be seen. This occurs in the case of industry informant TBI-1, who mostly appeared to ignore any tack or any factors that could be considered impacting on the horse. TBI-1 also avoided descriptions of any mental or behavioural expressions of the horses. For example, in the case of the same Image 3 that elicited the most horse-centred response of any industry informant (TBI-9, Section 4.2.1), informant TBI-1 simply stated: “The horse is being led somewhere, probably to the gate”.

Image 4 is the only image that elicited comments on the tack by all but one industry informant. They comment on the tongue-tie, and many responded similar to TBI-8: “He’s got a lot of equipment on”. TBI-3 and TBI-5 added the tongue-tie is very tight. The exception here is, again, TBI-1, who did not refer to the tongue-tie (but mentions the bit). While this is a passive downplaying through the act of ignoring, active downplaying is also evident. For example, referring to Image 3, TBI-4 acknowledged that “some horses are often agitated by the gate”. TBI-4 went on to explain that “it’s quite claustrophobic” and suggested other horses already in the stalls might be restless, banging the gates, jumping forward too soon or leaning back on the gate, and “there is a lot of noise”. This is one of the few instances where negative impacts were named and described by an industry informant. However, they were immediately downplayed by explaining it could be worse: “You know, no one has a stock whip on him, no one is hitting him, no one is, they are just trying to sort of coax it into the gate” (TBI-4).

#### 4.2.3. A Visual Problem and a Call to Educate the Public

In terms of Image 2, industry informants did not raise any welfare concern, as TBI-8 stated, “His ears are forwards, he doesn’t seem to be unhappy”. Instead, as TBI-5 explained, it is a problem with the “visual”, because people do not “really understand what is going on there”. This view became even clearer when TBI-5 responded to Image 4 stating, “The tongue-tie is a visual I have always struggled with. [...] The public sees a tongue-tie, [and] they want to know what that is. I understand the why and what [...] I am not a fan of it. I think it is an unattractive visual and I wish we had a better way of doing things there.” TBI-5 was not opposed to the practice as such; instead, the informant “really would like to find a better way of tying tongues” (TBI-5).

#### 4.2.4. The Thoroughbred, a Willing Participant

Industry informants tended to use positive terms when describing the thoroughbreds’ responses. This is particularly evident in relation to Image 1, where they said the horse is “on his toes”, “a bit fiery” and “pretty spirited”. They pointed to the readiness and excitement of the athlete in competition, comparing the horse to the human athlete and describing the thoroughbred as a willing, anticipating and knowing participant: “The horse is anxious, it’s a bit fiery, it’s business time” (TBI-3), and TBI-9 saw “a horse that wants to race” and added “I think horses know that they are going to race and they get excited.” Likewise, in relation to Image 2, industry informants saw “nothing out of the ordinary” (TBI-6), it is a thoroughbred who “wants to go and the jockey says ‘not yet buddy’" (TBI-5). 

### 4.3. Themes Emerging from Animal Advocacy Informants’ Photo-Elicited Responses

Four key themes also emerged from the animal advocacy informants’ responses to the photo-elicitation component of the research.

#### 4.3.1. The Thoroughbred under Stress, Anxiety, Being Agitated and Disturbed

Animal advocacy informants generally used terms pointing to a somewhat distressed state of the horse. In Image 1, they saw a horse who is “stressed”, “reflecting anxiety, a bit of nervousness”, “disturbed in some way”, “spooked”, “fighting the bit” and the word “agitated” was used several times. The descriptor “stress” was used frequently in relation to the other images. There were degrees of difference in interpreting the signs of stress. For example, in relation to Image 3, some described the horse’s action as “pulling back” (AAI-1), being “scared of where it is supposed to be going” (AAI-1) and “somewhat agitated” (AAI-6), but two advocacy informants did not regard the situation as acute when they stated the horse “isn’t rearing or anything like that” (AAI-3), and he “doesn’t look like he is in a major panic” (AAI-5). 

#### 4.3.2. A Wide Range of Factors and Unnatural Conditions Impacting Thoroughbred Welfare

While industry informants made limited mentions of the impact and role of tack and other environmental factors, animal advocacy informants saw a horse who is confronted with and impacted upon by many factors. While industry informants naturalised and normalised the flow of events they saw in the images, animal advocacy informants saw the denaturalisation of the horses’ environment and the use of particular practices and tack as impacting the horses negatively and as being a welfare issue. For example, in Image 2, animal advocacy informants saw a horse who is held very tightly and a bit being “pulled very severely” (AAI-2). They saw a throat lash that was too tight (AAI-4) and “don’t like that bottom ring on the bit” (AAI-5)”. They saw a horse with neck tension (AAI-5), a head “quite tucked in” (AAI-3) and a horse who is “very uncomfortable” (AAI-7). 

In contrast to industry informants, animal advocacy informants noticed more detail in the horses’ mental and behavioural expressions. For example, commenting on Image 1, more advocacy than industry informants referred to the horse’s movement, often describing it as “quick”; they referred to the flared nostrils, and five of the seven referred to the open mouth and, in one instance, to the tongue and to “pressure on its mouth” (AAI-2). Moreover, in relation to Image 1, no industry informant commented on the tack; however, five of the seven animal advocacy informants did so, all in negative terms as causing discomfort and pressure and contributing to an already “stressful environment for the horse” (AAI-4). AAI-3 stated “The other thing that really strikes me is how tight the bit is in the mouth”. AAI-4 explained the bit “looks like a Dexter ring bit [...] a very harsh bit” that causes the horse to resist; as AAI-4 stated, the horse appears to be “fighting the bit”.

All animal advocacy informants described in all images compromised welfare or the potential for compromised welfare. The following response of AAI-4 to Image 4 demonstrates the array of concerns identified from a perspective where horse welfare and protection is centred. The comments range from physiological to mental aspects, to hinting at the psychology of handling horses and racing regulations. While the breadth of concerns is not paralleled by any other advocacy informant’s response, this quote is illustrative of animal advocates’ concerns:

“Astounding. Absolutely astounding that this can ever be allowed. Which is, where the industry who talk about welfare of horses being a priority, this picture shows how bad the welfare is for horses. [...] [This horse is] absolutely stressed to the maximum. We see absolutely an overkill in the bitting and bridling of this horse. Again, we have the Dexter ring bit, which is a very severe bit for a hard-pulling horse. We’ve got a tongue-tie in there, which is obviously- We can only presume the agony for the horse. [...] We’ve got the horse with its mouth open trying to fight all that and [trying to get away] from it, which he can’t. We’ve got [...] a sheepskin noseband on there [...] to keep the horse’s head down. We’ve got a lead rein or a martingale coming off that Dexter bit [...]. His head looks beyond the vertical, so he has got airway obstruction. He has got three bits in his mouth. The nuchal ligament in the neck, he must be in agony with all this. You know the ligaments at the back of the neck, [...] they must be really stressed from all this, and probably, he’s got windpipe damage as well with all that going on. So, total overkill by people who do not understand this horse whatsoever. They are looking to control a horse through bitting and bridling that doesn’t want to be controlled. And this is welfare at its very worst. It’s a great photo to show that.” (Animal advocacy informant AAI-4)

AAI-4 is the only informant who referred to the conformation of the horse and its welfare relevance in racing. For example, in relation to Image 1, the informant described how compounding factors of horse conformation, tack and the way it is applied impact welfare. AAI-4 explained the horse has “a thick neck through the gullet, making flexion very difficult [...]. When horses have this conformation”, the horses “pull very strongly”. Consequently, “the trainer and the jockey [...] tend to put a stronger and stronger bit on the horse, trying to control the horse. And the more you do that, that exacerbates the problems [...]” Relating to Image 2, the informant added “That bit in the mouth is [...] totally wrong for this horse. [...] The parotid gland between the jaw and the atlas vein in the head [...] is very swollen, and that is bound to be painful.” Overall, “the cheek piece is in the wrong angle, and the throat lash looks very tight. [The horse’s] conformation [is] not suitable for racing at all, I wouldn’t think” (AAI-4).

#### 4.3.3. A Visual Problem Reversed, and Another Call to Educate the Public

Some animal advocacy informants also considered the public’s perspective but in a different light than an industry informant would (compare to Section 4.2.3). They agreed that the public does not understand what they see, if they saw it at all. As AAI-2 said in relation to Image 4, “you don’t often actually see what [the tongue-ties] look like quite in the way that this photograph depicts, and I think that’s a shame, because if people knew what a tongue-tie was and the effect that it had on the horse, they perhaps wouldn’t allow them to be used”. AAI-2 added that this is “just about as unnatural as you can get, going back to the word natural.” Likewise, AAI-1 pondered: “I don’t expect that most people, either at the track or elsewhere, would see this, meaning be able to see it or understand what they were seeing. Or understand that this is not a natural thing for horses, this is something imposed by the industry.” This contrasts with the perspectives of the industry informants, who, as TBI-5 stated, would prefer a less visible device to tie the tongue, so the public does not see it.

#### 4.3.4. Horse-Human Interaction

Animal advocacy informants took more notice of the presence of the depicted humans and the impacts they have on the horses than did industry informants. In relation to Image 1, five of the seven advocacy informants referred to the human and her handling of the horse. They stated, it “looks like she is having to really focus on handling that horse” (AAI-3), and she “is trying to calm down a very excited horse” (AAI-6). Emphasising the presence of the handler and her action support the perspective that the horse displays mental and behavioural expressions to a degree and at a severity that require intervention. AAI-4 believed the handler contributes to the horse’s stress, because the horse is on a “very stressed rein” and resists the bit. In contrast, four of the nine industry informants referred to the handler, but the description of the human’s presence and her interaction with the horse was minimal. Mostly, the handler is somewhat absent when simply stating the horse “looks like saddled in the paddock [mounting yard]” (TBI-1). TBI-7 is the only industry informant who described a more aggravated situation, stating the handler “is trying to do her best to manage the horse”. 

Commenting on Image 3, animal advocacy informants described in more detail the presence and the actions of the handlers. Many saw “an awful lot of people” (AAI-2), “helmeted people” (AAI-1), contributing to the stress they believed the horse was already experiencing. They used terms like “force” (AAI-4, AAI-3) applied by handlers and people “pulling” and “dragging on the bit with a lead rein or rope” (AAI-4). AAI-4 also noticed that, while the jockey does not show signs of stress, the handlers do, and “that is impacting on the horse and he is planting himself.” Moreover, while advocacy informants saw humans acting on the horse, AAI-2 went a step further, describing a lack of engagement with the horse at the level of the horse, with no attempt to respond to the horse sympathetically in a way that allows two-way communication. AAI-2 observed the handlers “are not focused on the horse at all, none of them are looking at the horse’s face. None of them are really looking at the horse other than holding on to the saddle or just intent on moving it somewhere”.

There are two negative cases present (one in each group) in relation to Image 3. In contrast to other animal advocacy informants, AAI-7 was unconcerned: “It looks like [...] the horse is alerted to its surroundings and perhaps looking at other horses or something ahead.” On the other hand, and in contrast to the other industry informants, industry informant TBI-9 shared the concerns for the horse with the advocacy informants (see Section 4.2.1). 

### 4.4. Conceptualisations of Naturalness and the Nature of the Thoroughbred

This section discusses the responses to the verbal-only interview questions of the current study and the earlier published results of the informants’ conceptualisations of naturalness [3], with reference to the photo-elicited responses. The interview questions asked the informants about what the thoroughbred represents for them, what they believe is the most natural (equestrian) activity for the horse and how they define the term naturalness (see Section 3.3). The results demonstrate that the informants have limited awareness of naturalness as a concept; however, their conceptualisations were inferred based on how they used ideas of naturalness and what is natural (see Section 3.3).

#### 4.4.1. Thoroughbred Industry Informants

The industry informants were not familiar with the concept of naturalness. Three of the nine informants volunteered to further engage with it when asked to define it or whether they have heard of it, two of them only after prompting [3]. The conceptualisations of all informants, however, could be inferred from their other responses. In the current study, contradictions emerge in the role nature and what is natural play between how the industry informants explained and justified racing practices and how they conceptualised the thoroughbred at the ontological level. Describing what the thoroughbred stands for, the industry informants focussed on the idea of the athlete, referring to “magnificent athletes”, “athleticism” and, as TBI-3 stated, “the extreme athlete of the horse world”. Some emphasised that thoroughbreds are bred to be athletes (TBI-4) and “bred for performance” (TBI-3). Thus, they appear to see the thoroughbred as a breed rather than a horse and differentiate them from other horses; TBI-8 poignantly described thoroughbreds as “the pinnacle of refinement of the equine species”. Overall, it appears the thoroughbred is considered to be an improvement on nature to a degree that they are somewhat separate from nature, and it appears there is some pride in this achievement. It is the thoroughbredness of the thoroughbred rather than the horseness of the horse (see also Bergmann [3]) that the industry informants seemed to conceptualise, a species somewhat different from the horse.

With one exemption, industry informants suggested mostly racing but, also, running or galloping are the most natural activities. TBI-4 added they “love to run, gallop, between the fences, on the beach, some even love to jump"; they love to “use their bodies in that way”, which was seen in contrast to dressage, which was described as “very controlled” (TBI-4). TBI-2 suggested racing is “the [activity] most aligned to one of the key instincts of the horse, which is to run in a herd”. Two informants referred back to the nature of the wild horse, as, for example, TBI-8 stated “anything that leverages of things that they would do normally in the wild is something that falls within that range”. This defence of racing as being natural is consistent throughout the industry at large. However, it ignores the difference between the horse’s self-determined or invoked turnout behaviour, on the one hand, and highly regimented training and racing practices, on the other hand (see also Section 1). The impression “horses love to run” is most likely based on horse behaviour that is in fact influenced by the unnatural conditions they are kept, which applies in particular to racehorses in preparation and training who are kept stabled. Horses in confinement react with increased activity when not confined [103]. Chaya et al. [103] found horses who were given only short turnouts during the day were more likely than those given longer turnouts to trot, canter and buck when turned out, thus displaying what is considered compensatory locomotor activity [103] (p.156). Similarly, Przewalski horses kept in smaller enclosures spent more time pacing and milling than the comparison group kept in a larger enclosure [40].

When referring to “key instincts” and what is natural, reference to the horse was made rather than the thoroughbred, again distancing the thoroughbred from the horse. The industry informants’ dominant narrative that thoroughbreds love to race and that racing is the most natural ridden activity for the thoroughbred (except in one instance, TBI-3) is consistent with their naturalisation of the thoroughbreds’ mental and behavioural expressions and racing practices (Section 4.2.1). It lends strength to their justification of the activity of racing and is consistent with the dominant approach of downplaying and trivialising what could evoke welfare concerns (Section 4.2.2). However, the thoroughbred’s ontological removal from nature is in contradiction to the industry informants naturalising the thoroughbreds’ mental and behavioural expressions on race day.

A lack of attention to the horse-human dimension also emerges from the responses to the verbal-only interview questions. Only one industry informant referred to the horse-human interaction, and this informant described what they considered to be a natural shared activity: “Horses and their owners or riders get a real strong bond, and there is nothing a horse enjoys more than being out on a ride or being groomed and set ready for activity. I don’t think it has to be racing” (TBI-3). The otherwise demonstrated lack of interest in the horse-human relationship corresponds with the industry informants’ tendency to ignore and downplay the presence and impact of the humans and their actions depicted in the images (Section 4.2.2 and Section 4.3.4). It seems the industry informants mostly did not consider the horse-human relationship a factor impacting welfare, let alone having a relational ontological presence [31,104] in its own right. 

The construction of the thoroughbred dominantly as an athlete and a breed, being bred for racing and loving racing, is not static. Two industry informants expressed views that also see the thoroughbred as a horse. For example, TBI-4 emphasised the thoroughbred is a social species who “love[s] to be in a herd”. The idea that thoroughbreds are individual in their personalities, strengths and weaknesses was also expressed (TBI-4 and TBI-7). Three other industry informants placed emphasis on the thoroughbred being “smart” and “trainable” (TBI-7) and “highly adaptable” for other “athletic pursuits” (TBI-2). TBI-1 added they are “also a very kind animal [epitomising] a lot of special qualities as an animal, as an athlete and as a companion”. The comments emphasising trainability and adaptability were made in the context of retirement from racing and the thoroughbred’s suitability for a life after racing. This ontological flexibility from the athlete, being purpose-bred and loving racing, to the trainable and adaptable athlete and companion was made by informants with a stake in thoroughbred aftercare (i.e., life after exiting the racing industry) and suggests there is a pragmatism and opportunism in conceptualisations of the thoroughbreds’ nature. It seems a reframing of their message had taken place, aimed at a particular audience, such as the researcher, those potentially interested in retired and retrained thoroughbreds and the public at large [105]. 

In summary, the industry informants remained distant from the concept of naturalness; they appeared to see the thoroughbred as a breed somewhat separate from nature and a species somewhat different from the horse. Nonetheless, they relied strongly on constructing a notion of the nature of the thoroughbred and of what is natural that defends racing practices. Their conceptualisations of naturalness were not only fragmented, contradictory and inconsistent but reductionist, instrumental and opportunistic according to their messaging needs.

#### 4.4.2. Animal Advocacy Informants

In contrast to most industry informants, all but one of the seven animal advocacy informants demonstrated great interest in engaging with the notion of naturalness, although most, like the industry informants, did not recognise the term as such [3]. This interest finds resonance in referring to the thoroughbred as, first and foremost, a “horse” or “animal”, rather than a “thoroughbred” or a “breed” wedded to racing. They described the thoroughbred as a “magnificent animal, powerful, strong but also sensitive” (AAI-3), a “fragile animal” (AAI-1) and, also, “possibly the most beautiful animal on earth” (AAI-6). AAI-6 also pointed out they are all “beautiful individuals”; “they all have individual needs, likes and dislikes, different temperaments”. 

However, most advocacy informants also described the thoroughbreds as animals who are highly exploited and deprived of their agency, as being placed at risk by human hands (AAI-1), as having a “less honourable connection with gambling and profiteering” and as a status symbol for humans (AAI-3). AAI-6 described the link between exploitation and deprivation:

“I also think of them as greatly exploited, because they have so little say in their lives, even those horses who are considered successful at what they do, there is usually no one person who is committed to that animal for their whole lives. They go off from barn to barn, they move from trainer to trainer, from jockey to jockey and all too often end up someplace horrible, at least in the United States. So, they are on the one hand the most revered, and on the other hand, the most discarded animal that I know of.” (Animal advocacy informant AAI-6)

This response exemplifies that the animal advocacy informants’ responses to the verbal-only interview questions carry mostly negative connotations when referring to the horse-human relationship in the context of the thoroughbred industry. This echoes their photo-elicited responses. Describing the images, they saw the humans doing something to the horses that was mostly seen as being against the horses’ interest and welfare. The exploitative dimension was, however, also presented by two advocacy informants (AAI-5 and AAI-7) in pro-economic and social-cultural terms when they referred to the thoroughbred as a breed of economic value and prestige, with impacts on the equine industry and the entertainment industry more broadly, with a global “trickle-down effect from the thoroughbred racing industry throughout the entire mainstream equine world and into other breeds, people and their desire to become involved with horses because of this” (AAI-7). 

Animal advocacy informants were mostly critical of the idea of referring to any ridden activity as “natural”. They suggested instead more horse-centred categories for what is natural, as AAI-4 stated, natural is only “grazing, go almost feral [...] The others are peripheral events [to] utilise a horse’s qualities [...] for transport, for leisure and for sport.” They suggested all activities exploit the horses’ abilities and not “any one is more natural to a horse than another” (AAI-6). AAI-2 affirmed “there is not a lot that is really natural about keeping domestic horses in any case. So pretty much everything we do, I don’t think you could describe as being natural”. They identified a broad range of factors that violate the nature of the thoroughbred, including many aspects of the overall environment. AAI-2 suggested any use of horses involves a range of activities that “are all issues in terms of welfare, all that is unnatural”, including removing the horses from their familiar environment and social group, transportation, confinement, the competition arena and mixing horses unfamiliar with each other. Some suggested, however, where there is a bond, a horse-human relationship, for mutual benefit, certain activities may be acceptable but not when the horse is forced to do something (AAI-3). Within this frame of reference, trail riding—not endurance riding, as one informant points out—is an activity for which some have some tolerance in terms of what is natural for the horse (AAI-1, AAI-5 and AAI-7). As AAI-7 stated, trail riding is what the horse would do in nature, “whether in the wild or domesticated horses in captivity, they like to run”. They generally referred to running rather than racing. 

In summary, the animal advocacy informants’ conceptualisations of naturalness and what is natural are consistent throughout their responses to the verbal-only questions and to the photographs. They demonstrated a more holistic idea of naturalness. They related naturalness to the many aspects of the thoroughbreds’ lives, to their natural emotional and behavioural needs, their telos, health and healing, husbandry and training practices and to how humans relate to them (see also Bergmann [3]). They related it to the thoroughbreds’ horseness rather than “thoroughbredness”, and based on this, they mostly argued that racing practices are not in the interest of thoroughbred welfare. They tended to recognise a denaturalisation of the horses’ life world, condition and treatment and a violation of their nature, integrity and agency. Overall, and in contrast to the industry informants’ conceptualisations, the animal advocacy informants’ ideas of what is natural are more consistent with ethological perspectives [40,41,42].

### 4.5. Naturalness as a Lens for Thoroughbred Protection

In the following subsections, the themes emerging from all informants’ responses are synthesised and discussed: Naturalising, normalising and downplaying racing practices and their impacts; the thoroughbred as an eager and willing participant versus a horse under stress, anxiety, being agitated and disturbed; the perception of equipment and its applications; the visual problem as a problem of showing too much or not enough; the horse-human relationship and the idea of the thoroughbredness of the thoroughbred versus the horseness of the horse. The themes are discussed within the context of research in relation to impacting factors that are raised by the informants—namely, the bit, the tongue-tie and human handling. Two examples of recent interventions from a well-known racetrack operator in North America and the Australian racing authority are included (see Section 4.5.2) to support the findings and illustrate the hermeneutic research approach (Appendix A). In Section 4.5.4, Bergmann’s Layers of Engagement with Animal Protection [3] are applied to deepen the analysis of the thoroughbred welfare and protection discourse. Recommendations for further research conclude this section (Section 4.5.5).

#### 4.5.1. Naturalness as a Guide Versus Naturalness as a Fallacy

What seems to be a significant factor in the industry informants’ process of naturalising, normalising and downplaying racing practices and their impacts on the horse is that many such practices exist because they have “always been done that way”. In the case of bits, for example, Mellor and Beausoleil [17] find that most horses “exhibit clear behavioural evidence of aversion to a bit in their mouths, varying from the bit being a mild irritant to very painful” and believe that this in itself is a significant welfare issue requiring attention [17]. They suggest “the non-recognition of clear behavioural evidence of horses’ aversion to bits in their mouths arises because the indicative behaviours have been and are observed so commonly that, except in more extreme cases, they are considered to be normal” [17]. Cook and Kibler [20] (p. 551) suggest that, because bits have been standard equipment for millennia, they “are widely assumed to be indispensable and ethically justified”.

When calling on what is natural, one can be expected to question what really is natural. If naturalness was a guide, a starting point to assess the expressions of the thoroughbreds in the images and elsewhere could be similarity to the “closest wild counterparts” [48] (see also Section 2). In the case of the bit, Cook [21] (p. 256) summarises: “At liberty, the running horse has a closed mouth, sealed lips and an immobile tongue and jaw”. The horse is an obligatory nose-breather, and the application of a bit breaks the seal of the lips [106]. This has a raft of implications for health, welfare, ability to perform and safety, including bit-induced pain being a cause of fear, flight, fight and facial neuralgia, the bit interfering with breathing and locomotion, the bit being implicated in breakdowns and fatal accidents, and it is hypothesised that the bit causes dorsal displacement of the soft palate, induces asphyxia, which causes bleeding from the lungs (EIPH), and it can cause sudden death [21,106,107]. Moreover, Mellor and Beausoleil [17] conclude that the bit impacts horses in a way that they experience severe breathlessness. 

Instead of questioning the application of the bit, the industry informants saw it as part of a normal and natural system in racing. For example, Image 2, which depicts the head of a ridden-bitted thoroughbred with an open mouth identified by Mellor and Beausoleil [17] as a sign of aversion to the bit, was described by industry informants as depicting “nothing out of the ordinary” (TBI-6), showing “actually a very gentle bit” (TBI-4), and TBI-4 explained that the mouth opens not because the jockey is “tearing at his mouth” but because “the horse is wanting to go forward”, and, so, “the horse [...] is pulling against his mouth”. Most industry informants also expressed support for the use of added pressure-exerting tools and practices to deal with the problems the application of the bit and training, racing and handling practices cause, such as the use of yet harsher bits and nosebands and the application of the tongue-tie (Section 4.2.2), despite their welfare implications and lack of efficacy [19,108,109,110]. Other practices in the industry at large, to address health and performance issues, potentially linked to use of the bit [21,106,107] include use of the contested drug furosemide [111] and surgery performed at the horses’ upper respiratory tract [112,113,114]. These are common interventions despite the side effects of the drug furosemide [111] and the potential for complications as a result of surgery, with subsequent health and welfare implications for the thoroughbred [115,116,117,118]. The central focus of these interventions is generally not to protect thoroughbred health and welfare but for humans to pursue an activity that pushes the horses beyond their natural physiological limits. Indeed, those involved in the care of racehorses identified the overuse of veterinary interventions as a significant welfare challenge [61].

The examples discussed above demonstrate how calling on what is natural can be a fallacy when divorced from scientific evidence and from the horses’ interest in their own physiological and psychological integrity. It also demonstrates how naturalness as a guide is relevant for thoroughbred welfare and protection even in an environment and under a handling and exercising regime that controls all aspects of their lives and has significantly compromised their nature, agency and integrity.

#### 4.5.2. Naturalness and the Legitimacy of Thoroughbred Racing

Naturalising and normalising the horses’ emotional and behavioural expressions and the impact of particular racing practices depicted in the images can be seen as an attempt to legitimise racing. There are indications that the industry informants were aware that the thoroughbreds’ expressions can be perceived as compromised welfare, as TBI-5 expresses concern about the visual of the tongue-tie (Section 4.2.3), and TBI-4 adds, when commenting on Image 2, that the open mouth is “not a pain mechanism”. The industry informants’ tendency to ignore and, thus, conceal potential welfare concerns embedded in common racing practices as a way of addressing the public’s perception of racing appears to be an approach taken throughout the international racing industry. For example, The Stronach Group’s media department reportedly has specific instructions to reduce the use of images showing certain whip actions in racing [119]. In 2018, the Stronach Group’s Gulfstream Park racetrack even produced and distributed a promotional wall calendar that reportedly contained images with some of the whips carried by jockeys in the racing action shots digitally removed [119]. In at least one instance, not only had the whip been removed but the bit had also been digitally altered to appear as less severe than in the original photograph (see the original and the manipulated images on pp. 5,6 in the article written by T.D. Thornton for the Thoroughbred Daily News [119]). The tendency of the industry informants to not put into words the extent of the mental and behavioural expressions of the horses, and the impact of the equipment used or the human handling of the horse (Section 4.2.2), functions similarly to how digital image editing tools are used as a way of “unseeing” what they prefer not to be seen. The industry informants presenting certain aspects as normal and natural indicates they are consciously and subconsciously participating in the industry’s priority project to change and shape the public’s perception of the racing industry and its treatment of the thoroughbred, a phenomenon that can also be observed in other equestrian disciplines [120]. 

What TBI-5 identified as a visual problem is a problem of legitimacy of the horseracing industry [39,121]. With their attention directed at sanitising the visual, the industry engages in censorship and resists transparency. This undermines trust in the industry, and trust is an indispensable aspect of legitimacy [121]. The industry is aware of the risk to its social license to operate [121]. Nonetheless, in particular racing in the UK, Australia and the US, the regulating racing bodies are resistant to centre the protection of the thoroughbred over industry interests. In Germany, German Racing banned the use of tongue-ties as Rüdiger Schmanns, then Director of Racing for German Racing, stated “[w]ith growing animal welfare activities, especially in Germany, there was no possibility of allowing the use of tongue ties to continue” [122]. In 2020, Racing Australia reaffirmed their position that the tongue-tie is acceptable, arguing they have found “an appropriate balance between the welfare of the horse and performance” [123], despite its disputed efficacy and need [124] and health and welfare impact [19,125]. 

The application of the bit and the tongue-tie are but two examples. Butler et al. [61] identified a raft of welfare issues and challenges that demonstrate how common racing practices put thoroughbred welfare at risk. It can be expected that the racing industry will come under increasing pressure if more details of their common practices in racing—and breeding thoroughbreds, for that matter—become increasingly known to the general public. This is largely due to the implications for thoroughbred welfare and the nature of the horse and the concern people show for naturalness in determining what a good life for an animal is [46,52,58]. Currently, industry representatives take the view that the problem is not the impact of racing practices on the horse but that people do not “really understand what is going on there” (TBI-5, see Section 4.2.3), an aspect previously discussed by Bergmann [2] (pp. 127–128). Indeed, many people are unaware of the common handling and training practices in racing, and animal advocates believe there is a need to inform and educate the public. Referring to the tongue-tie in Image 4, advocacy informant AAI-1 did not “expect that most people, either at the track or elsewhere, would [be able to] understand what they were seeing”. However, a lack of public awareness cannot be used as an excuse to continue to harm thoroughbreds, nor as an “excuse to ignore the unrepresentative nature of existing welfare policy” [46] (pp. 29–30). For welfare policy to have democratic legitimacy, it needs to reflect the public’s view of what it means for a nonhuman animal to fare well [46].

#### 4.5.3. The Horse-Human Relationship as an Aspect of a Holistic Notion of Naturalness

In the responses of the animal advocacy informants, the horse-human interaction emerged as an important element for horse welfare (Section 4.3.4). This echoes Butler et al. [61], who found that the horse-human relationship was identified by those professionally caring for thoroughbreds as a seminal aspect of good welfare. The participants referred to factors such as the “consistency of routine and carer” and horse and human “getting on”, ensuring continuity and attention to detail and not only well-trained and knowledgeable but experienced staff for a “best-life” scenario. Creating a positive horse-human contact was linked to a potentially higher level of care and observation. Hall et al. [11] described the link between human handling and horses’ emotional and behavioural expressions:

Horse-human interactions undoubtedly influence both the subjective emotional experience and the behavioural expression of the horse. The influence may be due to the intensive management, handling and focused interaction associated with the process of training, and the physical and emotional demands placed on the animal in relation to performance. Methods of training and handling which provoke negative emotions and states such as fear, or where the individual experiences pain, may lead to short term success in relation to behavioural change, but will also produce fearful horses which are not desirable for the horse or human safety, nor successful for performance in the longer term. When frightened or anxious, horses will show escape responses ranging from agitation involving a raised head and neck to extreme reactions including bolting [11] (p. 184).

Most industry informants ignored or downplayed the human factor in the images, including in Figure 3, depicting a thoroughbred resisting to enter the starting gate. This may be a result of the informants interested in conveying to the researcher that there are no welfare issues to be seen. It could also be a case of nonrecognition, as discussed in the context of the bit above, due to the normality of horses expressing fear and resistance at the starting gate. As Miles et al. [25] found, 71% of the studied 2–5-year-old racehorses entering the starting gate demonstrated “unwanted” behaviours. They also found that gate staff responded by using an “artificial aid”, such as whipping over 40% of the time, which explains why TBI-4 made the downward comparison in relation to Image 3, stating “no one has a stock whip on him, no one is hitting him” (Section 4.2.2). Moreover, it can be suggested that many of the emotional and behavioural responses of the thoroughbreds in the images may, in fact, be learned or shaped by the human factor and the particular activity of racing as such [24]. The kind of relationship humans have with the horse shape the nature of the handling and training practices, and vice versa, the handling and training practices shape the nature of the horse-human relationship. It is suggested that the underlying horse-human relationship plays a significant role in how the human and how the horse respond [11]. The low interest in the human-horse relationship and lack of recognition of its importance for equine welfare is characteristic of the industry at large. The participants of Butler et al.’s study [61], for example, identified staff shortages and a lack of experienced staff as a challenge significantly impacting thoroughbred welfare in various ways. 

For a better understanding of the horse-human relationship, this author suggests contextualising it within the framework of naturalness. This contrasts with Yeates [48], who believes other animals’ interactions with humans are unnatural, and therefore, human-animal relationships are not an aspect of naturalness. However, humans have lived for tens of thousands of years in multi-species communities, whether in close proximity or not. Therefore, it seems more useful for animal protection in a multi-species world to conceptualise human-animal relationships and interactions as being an aspect of naturalness. A reductionist approach to naturalness and the human-animal relationship would mean to artificially separate the innate connection between humans and other animals that is based in a shared evolutionary continuity, also expressed as kinship [126]. The argument is based in the binary of humans versus nature and the belief that humans are separate from nature is considered by many one of the root causes of human exploitation of animals and nature [127] and is counterproductive to advance animal protection. The question is, rather, what human-animal relationships should look like under a framework where naturalness is intrinsically valued. Investigations in, for example, fields such as cognitive ethology [128] and into the ontological nature of the human-animal relationship [31,104] can assist in finding answers.

The welfare impact and the ontological status of the horse-human relationship discussed above speak to a definition of naturalness as a holistic notion. The raft of day-to-day welfare issues identified in the general equine welfare literature and unified by the notion of naturalness (Section 1), the many aspects of an animal’s life in which people relate to naturalness when thinking about a good animal life (Section 2), the role of naturalness for many equine welfare issues identified by particular groups of horse people, such as owners/riders and others involved in the care of horses [59,60,61], and the animal advocacy informants’ conceptualisations of naturalness (Section 4.4.2) all highlight the holistic qualities of the notion of naturalness. It appears that reducing this concept to one or a very limited number of aspects is arbitrary and an opportunistic reconstruction of its generic meaning. When narrowing down the meaning of naturalness to this degree, a different term that more accurately reflects what is referred to, such as natural nonhuman animal behaviour only, rather than naturalness should be used. A reduction obscures and co-opts the notion of naturalness and serves the user of the animal rather than the animal’s full range of interests and needs. Accordingly, industry informants dominantly use the concept of naturalness selectively when it aligns with their economic model (of breeding) and their activity (of racing). 

#### 4.5.4. The Layers of Engagement with Animal Protection and Naturalness

Previous research that explored the interface of thoroughbred welfare and sustainability found that the industry informants are, in some ways, the progressives in the industry, and they are situated at the reform level of the industry’s welfare discourse [3]. This current research, however, highlights that there are few individual cases where industry informants share similar concerns to advocacy informants (for example, TBI-9 responding to Image 3, Section 4.2.1). In this research, the informants were given the opportunity to defend the horse and reconsider current practices based on the images presented (see Section 3.3.1). However, when it comes to the handling of horses and the application of equipment, the industry informants appear to be more interested in defending current racing practices and maintaining the status quo (Section 4.2). This bears significant ongoing risks for thoroughbred welfare and protection.

**Figure 6:**
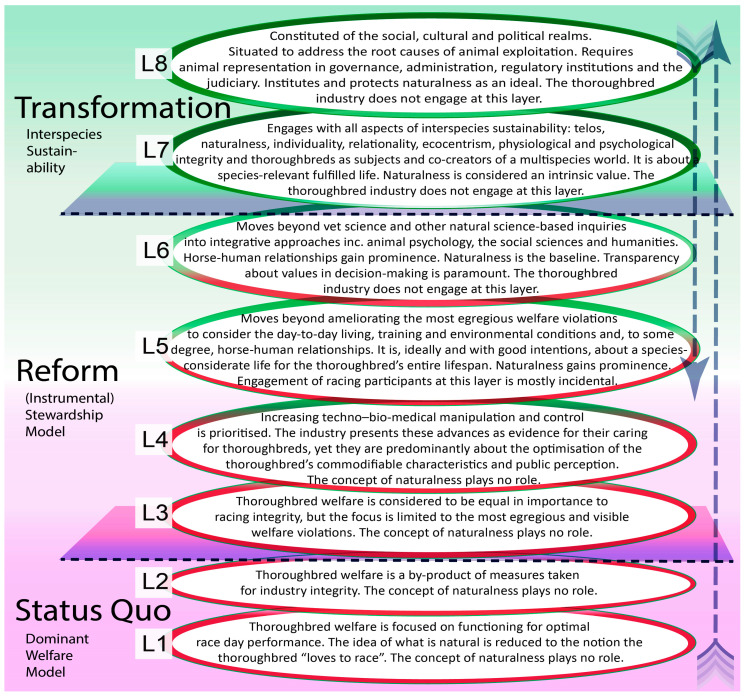
Layers of engagement with thoroughbred protection and the concept of naturalness. Indicates the status of the concept of naturalness within the discourse as described by Layer 1 to Layer 8 (L1–L8). The status of the thoroughbred industry discourse is situated within each layer.

The framework of Layers of Engagement with Animal Protection [3] is applied to further analyse and discuss these findings. Figure 6 is a further development of the layers presented previously in table format (see Table 5 in Bergmann [3]) to incorporate naturalness in more detail. 

Eight layers were identified. They range from those layers striving to maintain the status quo (Layers 1 and 2) through reform (Layers 3–6) and to those aiming at transformation (Layers 7 and 8). There is no strict separation between the discourse affiliated with any layer. The discourse on a particular issue can move up and down these layers, and the layers can overlap. The layers are not necessarily exclusive but can be, and any of the layers can be engaged within a discourse concurrently. They can augment each other but, also, be contradictory and difficult or impossible to reconcile. It is important to be aware of at what layer(s) the discourse takes place. The layers were identified in the context of the thoroughbred racing industry, but they can be adapted to interrogate other animal industries, interspecies activities or multi-species communities.

Most industry informants’ comments explaining and justifying racing practices invoking the natural take place at Layers 1–4. At these layers, the discourse focusses on functioning for optimal race day performance, with welfare being a by-product of or equal to integrity measures. The industry informants’ discourse supporting techno-bio-medical control (Layer 4) is prioritised to optimise the commodifiable characteristics of the thoroughbred. At the same time, these interventions were presented as being in the interest of thoroughbred welfare and safety, as, for example, TBI-6 and TBI-7 responded to Image 4, the tongue-tie is for the safety of the rider and horse. Thoroughbred welfare, as such, gains more weight in the industry discourse at Layer 3, where the focus is on the visible and most egregious welfare violations [3], but the idea of naturalness is irrelevant at that layer, as it is for industry integrity, at least from the industry’s perspective (more on the discourse in the intersection of industry integrity and racehorse welfare in Bergmann [3]). Concern for naturalness was reduced to the legitimating rhetoric that the horse “loves to race”. At Layers 1–4, the industry informants and the thoroughbred industry at large see nature as a limiting factor to be overcome through invasive means such as breeding (Section 4.4.1), the use of drugs (such as furosemide), surgery and equipment (see Section 4.5.1). 

Layer 5 offers opportunities for significant engagement with naturalness with its interest in the day-to-day living, husbandry practices, training and environmental conditions and, to some degree, horse-human relationships and the consideration of the horse’s entire lifespan. Here, the general animal welfare discourse places at least equal focus on the day-to-day conditions while centring the horse, thus potentially preventing many of the egregious welfare violations. Five industry participants (Section 4.4.1) made reference to aspects of Layer 5 to varying degrees, including interests in retraining and rehoming retired racehorses, thus acknowledging the natural lifespan of the thoroughbred extends beyond their use in racing and breeding and that this should be catered to. This interest in aftercare, however, is largely due to public concerns and animal advocacy campaigning and, at this point in time, appears confined to reaching for “low-hanging fruit” projects, signalling that the industry is responding to welfare concerns of “wastage” [129]. There is, however, potential for the discourse around aftercare to move beyond Layer 5 as developments in aftercare evolve, as the discourse around human-animal relationship develops and the protection status of nonhuman animals grows.

Where Layers 5 and 6 meet, the horse-human relationship gains relevance in the discourse. When discussing naturalness, one industry informant (TBI-3) related to the horse-human bond in one instance (Section 4.4.1). Generally, however, at the systemic level, Layers 5 and 6 currently have limited relevance for the industry informants and the industry at large. At Layers 5 and 6, the discourse moves beyond veterinary science and others based in the natural sciences. Layer 6, in particular, is situated in the scholarly discourse to engage with, for example, (noninvasive) research in animal welfare, ethology, equitation science and the social sciences. Yeates [48], for example, can be said to be engaging with naturalness at Layers 5 and 6, but the limitation placed on his definition of naturalness as relating to natural animal behaviour only and being distinct from species-specific needs [48] limits its potential for advancing into broader animal interests and the discourse taking place at Layer 7. It can be expected that those in racing engaging at Layers 5 and 6 will inevitably sooner or later engage more with the concept of naturalness. This is confirmed with the description of the “best-life” scenario for a racehorse in Butler et al.’s study [61], where the discourse of the “best-life” scenario takes place at Layer 5 and, to some degree, at Layer 6, with the study participants emphasising a positive horse-human relationship and aspects of naturalness. In the interest of thoroughbred welfare and protection, there is a need to shift the focus onto the horse-human relationship as a welfare issue in racing while the industry exists.

It appears that, in contrast to industry informants, animal advocacy informants overall had a strong interest in engaging with Layer 5—in particular, with aspects of naturalness. Some also engaged with aspects of naturalness at Layers 6 and 7. How the animal advocacy informants of this study conceptualised naturalness resembles how people in general consider naturalness. Both tend to view naturalness in holistic terms, including a variety of considerations (Section 2, Section 4.3 and Section 4.4.2). 

Industry informants did not engage with Layers 7 and 8. These are the layers where a holistic notion of naturalness plays an essential and defining role for animal protection. Naturalness is considered an inherent worth to be protected and preserved. A rethinking of the ontological status of the thoroughbred—to acknowledge the horseness of the horse (telos)—is also a hallmark of these layers. This goes hand-in-hand with recognising the essential status of naturalness based on evidence. Adopting a holistic notion of naturalness is expected to maximise its potential for thoroughbred protection. Furthermore, the recognition of the thoroughbred’s nature has to extend to a recognition of their individual natures. It has to go beyond the species to acknowledge the individual’s temperaments, preferences, abilities and boundaries; as one of the animal advocacy informants (AAI-6) stated, the horses “are not all machines who despite their pedigree and their backgrounds want to [...] race” (see also [3]). Engaging with Layers 7 and 8 aims at facilitating a fundamental shift in human attitudes, belief systems and paradigms. It moves toward engagement with animal protection on the animals’ own terms and implements structures and processes for animal representation. 

It can be expected that sections within society are interested in engaging with the notion of naturalness as an intrinsic value once the discourse at Layers 5–8 advances in society at large. This will have implications for how thoroughbred racing and breeding will be perceived.

#### 4.5.5. Limitations and Recommended Research

A limitation of this research is the relative lack of participation of industry informants from countries other than the US and Australia (see Section 3.2). A broader international participation would have been desirable. However, most of the informants are active at the international level and all play a key role in racing, with all holding senior level roles. Furthermore, in terms of numbers, the US and Australia belong to the top racing nations internationally [130]. Future research could aim at recruiting informants from other racing nations. In terms of animal advocacy informants, the number of organisations to contact was limited, and their representation can be considered satisfactory (see Section 3.2). Two other proposals for further research are presented below. These arise from the issues surrounding the horse-human relationship as it manifests in shared horse-human activities and from the impact of common practices on the thoroughbred as discussed throughout this article and, in particular, in Section 4.5.1, Section 4.5.3 and Section 4.5.4. 

The question arises as to how horse-human shared activities should look so that they increasingly align with Layers 6–8 as the thoroughbred protection discourse advances. Interest in the nature of horse-human shared activities is increasing generally [31,32,104]. The starting point for these considerations is the finding that, while some advocacy informants felt a sense of unease and violation arising from the horse-human interactions observed in the images, they still had some tolerance for horse-human shared activities (Section 4.4.2). This tolerance is conditional on the following: The shared activities should be within the realm of what is considered natural for the horse, they should provide mutual benefit for horse and human and they should not exploit the horse (Section 4.4.2). Framing research into the nature of shared activities within a naturalness paradigm is expected to assist in articulating what such shared horse-human activities that are ethical, nonexploitative and of benefit for the horse could look like. Re-evaluating the activity of thoroughbred racing within this context is of public interest for the following reasons: Racing’s legitimacy is in question due to the nonrecognition of the welfare impact of common racing practices (Section 4.5.2). Furthermore, animal welfare is conceived of as a public good by some [131], and racing relies on the public as gamblers and visitors to fund their enterprise.

The starting point for the second proposal is the welfare implications of tack—in particular, the bit and the tongue-tie—and common handling practices (see, in particular, Section 4.5.1 and Section 4.5.3). The question arises whether, and if so, to what degree thoroughbreds during and post-racing engagement suffer a form of post-traumatic stress disorder (PTSD). Common physical injuries are often described by those interested in ex-racehorses [132,133], but there is also anecdotal evidence that supports the suggestion that ex-racehorses are left with emotional trauma [134]. The evidence presented in Section 4.5.1 and Section 4.5.3 appears to lend support to this suggestion. PTSD has been shown to occur in other animals [135,136,137]; yet, the condition described as PTSD is generally not used in the literature to describe the psychological state of thoroughbreds showing particular symptoms. Noninvasive research to investigate the status of thoroughbreds in the context of PTSD and strategies to prevent its occurrence are required, as long as racing persists. This study has demonstrated that naturalness as a guide centres thoroughbred welfare and protection. It is therefore recommended to frame the suggested research within this paradigm.

## 5. Conclusions

This study has found that how naturalness is conceptualised is linked to how the impact of common racing practices on the thoroughbred are perceived and that this has direct implications for the welfare of thoroughbreds in racing. The current research has demonstrated the potential of the adoption of the concept of naturalness as a guide for thoroughbred welfare and protection that is adaptable to other interspecies activities, other animal industries and multi-species communities. There are indications that the welfare discourse is moving toward greater recognition of the concept of naturalness, and there is a potential for welfare policy and norms to shift more explicitly toward this notion as a signpost for a good animal life. Reducing naturalness to animal behaviour only limits its potential for animal protection. Instead, naturalness should be conceptualised holistically and as an inherent value of life, and the horse-human relationship needs to be recognised as a seminal aspect of naturalness.

Operationalising naturalness bears opportunities for the animal protection discourse. Applying the framework of the Layers of Engagement with Thoroughbred Protection and Naturalness can reveal when calling on what is natural and naturalness become fallacies. It assists in recognising the values and interests that guide or dominate the discourse and which conceptions are marginalised. It fosters transparency and assists in recognising whether the discourse is concerned with the protection of the animal or the facilitation of industry practices. As shown in this article, the Layers of Engagement can be used as a diagnostic tool to evaluate the discourse, to contextualise the intentions of those engaging in the discourse—is it reductionist, user- and industry-focussed or holistic and nonhuman animal-centred—to ensure advancing the interests of the thoroughbreds and other animals. Importantly, the model is adaptable so as to enable the interrogation of other interspecies activities, animal industries and multi-species communities.

In summary, the problems with thoroughbred welfare are much broader than the industry currently considers attention-worthy. The nonrecognition of the compromised health and welfare of the thoroughbred in racing resulting from common handling, training and racing practices poses significant threats to the thoroughbred and further questions the legitimacy of the thoroughbred industry. The industry will be increasingly pressured to address those issues with the discourse about common racing practices, animal welfare and naturalness advancing in society at large.

## Appendix A

### Trustworthiness

For trustworthiness, a number of procedures following Lincoln and Guba [138] were adopted. These include verbatim data transcriptions, ongoing comparisons between the analysis and raw data and the use of the informants’ own words when presenting the results. The conceptualisations of all informants in relation to the four images and the three verbal-only interview questions are presented. This includes negative cases [97] (pp. 309–313), which means here the presentation of cases that do not confirm the trend or the majority of the responses of a particular group of informants, and in relation to a particular aspect.

Different types of triangulation have also been applied. The two groups of informants were treated methodologically as two cases [139] and analysis was conducted within each case and across both cases. Comparing and contrasting the two groups’ responses addresses the credibility (validity), which is an aspect of trustworthiness in qualitative research [66] (p. 11). The employment of different data-collection methods (photo-elicitation and verbal-only interviewing) and the use of multiple theoretical perspectives from the natural and social sciences to explore and interpret the data increased the rigour and served the triangulations [140]. This means that the outcomes of the results obtained via photo-elicitation were compared with those obtained via conventional verbal-only interviewing. Finally, a deep hermeneutic approach [141] (pp. 560–561) was pursued—in particular, through the ongoing study of current events in the international racing context, including statements of industry bodies and racing participants cited in the media (two examples are presented in Section 4.5.2).
